# Parasite burden: Prevalence and risk factors in Ko-ae, Ubon Ratchathani, Thailand

**DOI:** 10.1016/j.parepi.2026.e00491

**Published:** 2026-03-02

**Authors:** Wirarat Jinatongthai, Phalakorn Suebsamran, Rerkchai Srivoramas, Tarinee Chaiwong, Jittiyawadee Sripa

**Affiliations:** a College of Medicine and Public Health, Ubon Ratchathani University, Warinchamrap, Ubon Ratchathani 34190, Thailand; b Research Group for Biomedical Research and Innovative Development (RG-BRID), College of Medicine and Public Health, Ubon Ratchathani University, Warinchamrap, Ubon Ratchathani 34190, Thailand; c Department of Civil Engineering, Faculty of Engineering, Ubon Ratchathani University, Warinchamrap, Ubon Ratchathani 34190, Thailand

**Keywords:** Parasitic infections, Risk factors, Health perception, Thailand, Cross-sectional study

## Abstract

**Objectives:**

Parasitic infections (PI) remain a significant public health concern in Southeast Asia. Understanding local prevalence and associated risk factors is essential for guiding effective interventions. This study aimed to determine the prevalence of PI and to examine the associated risk factors influencing PI in Ko-ae, a rural Thai community.

**Methods:**

A community-based cross-sectional survey was conducted in Ko-ae Subdistrict, Ubon Ratchathani Province, northeastern Thailand, from January to July 2021. Stool samples were collected from 941 participants and examined for intestinal parasites using standard parasitological techniques. Structured questionnaires were administered to assess participants' sociodemographic characteristics, health knowledge, and perceptions related to PI. Multivariate logistic regression was used to identify factors associated with infection.

**Results:**

Overall, 109 individuals (11.58%) were found to be infected with at least one parasite. The most prevalent single infections were *Strongyloides stercoralis* (4.14%), *Opisthorchis viverrini* (3.5%), and *Taenia* spp. (1.59%). Mixed infections involving both food-borne and soil-transmitted helminths were observed in 0.95% of participants. Male sex and older age were significantly associated with a higher risk of infection, while being employed and having a higher monthly income were protective factors. No significant associations were observed with body mass index, education level, or comorbidities. Participants demonstrated limited knowledge and inaccurate perceptions regarding PI. An increase in knowledge and perception scores was significantly associated with a 31% reduction in the odds of infection (odd ratio = 0.69; 95% confidence interval: 0.57–0.84).

**Conclusions:**

PI remain endemic in Ko-ae, with specific demographic and socioeconomic factors influencing infection risk. Knowledge and perception gaps were strongly associated with higher infection prevalence. Targeted health education campaigns, combined with chemotherapy interventions, are warranted to improve awareness, reduce transmission, and support sustainable control efforts in endemic areas.

## 1 Introduction

Parasitic infections (PI) remain a significant public health concern in many Southeast Asian countries, particularly in rural and agricultural communities. Despite extensive control efforts, numerous rural and urban areas in Thailand remain endemic for a variety of parasites (Boonjaraspinyo et al., 2022; Chuangchaiya et al., 2019; Punsawad et al., 2018; Sripa et al., 2025). Although the prevalence of PI has been studied in several regions, many areas remain underserved, and the limited availability of recent data on local epidemiology and behavioral risk factors continues to place inhabitants at risk.

Ko-ae, a sub-district of Khueang Nai located approximately 28 km from the city center of Ubon Ratchathani, a province in northeastern Thailand, is recognized as an endemic area for intestinal parasites. The presence of intestinal fluke larvae in freshwater fish and *Bithynia* snails in the rice fields of Ko-ae indicates fecal contamination and the distribution of helminth eggs and larvae in environmental reservoirs (Sripa et al., 2016). The geography, tropical climate, and presence of suitable intermediate hosts support the maintenance of parasite developmental stages and life cycles in this region. Additionally, the spread of helminth larvae to nearby agricultural zones, especially during the rainy season, facilitates transmission to humans and other natural hosts.

However, there is a lack of information on the prevalence and risk factors of PI in Ko-ae, as well as a critical absence of comprehensive data that integrates behavioral, socioeconomic, and cognitive variables associated with infection. In particular, the relationship between knowledge, perception, and infection status among inhabitants has not been investigated. Therefore, this study aimed to address these gaps by reporting PI prevalence and examining how sociodemographic characteristics, knowledge, and health perceptions influence infection risk. These findings may inform the development of targeted, community-based intervention strategies.

## 2 Materials and methods

### 2.1 Population and study areas

Ko-ae Subdistrict comprises 12 villages that remain predominantly rural, with extensive agricultural areas, including rice fields, gardens, and farms. It has a population of approximately 5872 and is surrounded by both natural water sources, such as swamps, ponds, streams, and creeks, and artificial sources, including weirs. Consequently, many areas in Ko-ae are located adjacent to these water sources, which serve as both food and irrigation supplies for agricultural purposes (The annual report on health services, Department of Health Service Support, Thailand, 2021).

### 2.2 Study design

This study involved a cross-sectional survey of PI conducted between January and July 2021 in eight villages of Ko-ae: Dong Yang, Na Mon, Na Pho, Tha Lat, Yang Noi, Pub, Ae, and Ko. The survey began with a one-month public announcement in each village to inform residents about the study's objectives, potential benefits, and risks associated with participation.

Village meetings were held in local community halls for participant registration. Research assistants provided all participants with an information sheet and verbally explained the study in the local language. Minors must have both parental and child consent before participating. For individuals unable to read, assistants read the information aloud, and informed consent was obtained via signature or fingerprint. Public health volunteers from each village served as witnesses during the consent process. Following this recruitment phase, 1573 residents from the eight villages consented to participate in the survey.

### 2.3 Exclusion and inclusion criteria

Participants were eligible for inclusion if they were permanent residents of the study area for at least six months and were aged 12 or older at the time of recruitment. Individuals were required to provide at least one adequate stool sample and/or to be able to participate in an interview or questionnaire, either independently or with assistance from a parent or guardian. Written informed consent was obtained from all adult participants, while parental or guardian consent, and child assent when appropriate, were obtained for minors. Individuals were excluded if they had received any antiparasitic or anthelmintic medication (including albendazole, mebendazole, praziquantel, ivermectin, niclosamide, metronidazole, or tinidazole) within the previous three months, as recent treatment could interfere with accurate detection of PI. Additional exclusion criteria included temporary residency in the study area for less than six months, severe acute illness or medical conditions preventing safe participation, inability or refusal to provide a valid stool sample, or cognitive or communication impairments that precluded reliable consent or data collection without a legally authorized representative. Individuals concurrently enrolled in other intervention studies targeting PI were also excluded to avoid bias in prevalence estimation.

### 2.4 Fecal sample collection

After registration, participants were provided with a fecal collection kit, which included a labeled screw-cap container (50 mm in height × 40 mm in diameter), a collection spoon, and an instruction sheet. A research assistant demonstrated the procedure for collecting fresh feces from rectum to avoid contamination with urine, soil, or water. Participants were given three days to deliver the collected samples to the community hall in their village. The fecal samples were then transported on ice each day to the Laboratory of the College of Medicine and Public Health, Ubon Ratchathani University.

### 2.5 Fecal examination

Fecal samples were processed using the gold standard method, the modified formalin-ether concentration technique (Anamnart et al., 2013). Briefly, 2 g of feces were resuspended in 10 mL of 0.85% NaCl. Debris was removed by filtering the sample through double-layered gauze into a conical tube. The filtrate was centrifuged at 2000 rpm for 2 min to obtain a fecal pellet. This pellet was then resuspended in 7 mL of 10% formalin and 3 mL of ethyl acetate to remove fats and residual debris. The mixture was shaken vigorously to ensure thorough contact between the fecal material, formalin, and ethyl acetate, followed by centrifugation at 2000 rpm for 2 min. The upper layer of fat and debris was removed using an applicator stick, and the remaining liquid was decanted. Residual liquid and debris on the tube walls were wiped off with gauze. The pellet was resuspended in 1 mL of 10% formalin, and the total number of drops was recorded. Two drops of fecal sediment were examined under a light microscope (Olympus, Model CX23LEDRFS1, Olympus Corporation) for the presence of helminth eggs and larvae, as well as protozoan cysts. The number of helminth eggs per gram of feces was calculated as follows: (number of eggs count/drop × total drops of fecal solution)/(grams of feces), to assess infection intensity (Laoprom et al., 2016). The intensity of helminth infection was classified as described by Laymanivong et al. (Laymanivong et al., 2014).

### 2.6 Drug treatment and questionnaire interview

Participants were individually informed of their fecal examination results. Only those diagnosed with PI received anthelmintic treatment according to the guidelines and at the discretion of a physician from Ubon Ratchathani University Hospital. Praziquantel (Bangkok Drug Co., Ltd., Thailand) was given in a single dose of 40 mg/kg for opisthorchiasis, and 10 mg/kg for taeniasis. Participants with taeniasis and severe constipation received a single-dose laxative. Albendazole (President Inter Pharma Co.,Ltd., Thailand) was prescribed as a single 400 mg dose for hookworm infection and at 800 mg/day for three consecutive days for strongyliodiasis. For giardiasis, metronidazole (Medic Pharma Co.,Ltd., Thailand) was given at 15 mg/kg three times daily for five days. Infection with *Entamoeba coli* did not warrant pharmacological treatment (Sripa et al., 2025). During treatment visits, research assistants also provided health education, including information on transmission pathways, diseases associated with PI and recommended hygiene practices to reduce the risk of reinfection.

Following drug treatment, a questionnaire-based interview was conducted to assess socio-demographic characteristics, knowledge, and health perceptions regarding endemic PI and cholangiocarcinoma (CCA). The questionnaire content was adapted from a behavioral control survey on PI and CCA developed by the Department of Disease Control, Ubon Ratchathani, Thailand. The language of the questionnaire was reviewed and modified to ensure clarity before the interviews were conducted.

Among the infected participants identified during stool examination, 80 individuals agreed to complete the questionnaire. To obtain a comparable control group, an equal number (80 individuals) of uninfected participants were subsequently recruited through random selection with the voluntary consent of the participants. Therefore, a total of 160 participants—80 diagnosed with PI and 80 without infection—consented to participate in the interview. Trained technicians conducted the interviews in the local language, and each interview was carried out privately.

### 2.7 Statistical analysis

Parasitological data were entered into Microsoft Excel. The prevalence of PI was expressed as the percentage of individuals infected with each parasite genus or species. Interview data were also entered into Excel and presented as percentages by questionnaire category. Descriptive statistic was used to analyze responses to knowledge and perception questions. The scores of knowledge and perception were calculated based on four key questions (Q1–Q4) reflecting awareness and preventive understanding of PI and CCA. Each correct response was scored as 1, and incorrect or “do not know” responses were scored as 0, yielding a total score range of 0–4.

All statistical analyses were performed using STATA version 18.0 (StataCorp, College Station, TX, USA). Associations between socio-demographic variables and PI status were evaluated using univariable logistic regression analysis. The odds ratio (OR), 95% confidence interval (CI), and *P*-values were reported. Statistical significance was set at *P* < 0.05.

### 2.8 Geographical analysis

The distribution of PI among participants in Ko-ae was analyzed based on household locations. The coordinates of participants' residences were recorded using a Global Positioning System device and subsequently mapped using a Geographic Information System program. Map markers were color-coded to represent different genera or species of parasites.

### 2.9 Ethical consideration

This study was conducted in accordance with the ethical principles outlined in the Declaration of Helsinki. All procedures involving human participants were reviewed and approved by the Human Ethics Committee of Ubon Ratchathani University (UBU-REC-46-2563). Participant samples were anonymized using coded identifiers prior to use for research purposes. All data files and records were securely stored in password-protected computers and locked filing cabinets, accessible only to authorized personnel.

## 3 Results

### 3.1 Prevalence of PI

Following registration, 941 participants submitted fecal samples for examination, resulting in a project participation rate of 59.82%. Among these, 109 participants (11.58%) tested positive for PI. Infections were more common in males (63.30%; 69/109) than in females (36.70%; 40/109). The highest infection rate among males occurred in the 41–50-year age group (26.61%, 29/109), while females were most frequently infected in the 31–40-year age group (17.43%, 19/109) (Table S1).

Among the 109 infected participants, 100 had single infections with either helminths or protozoa. The most prevalent parasite was *Strongyloides stercoralis* (4.14%; 39/941), followed by *Opisthorchis viverrini* (3.50%; 33/941), *Taenia* spp. (1.59%; 15/941), hookworm (0.63%; 6/941), and *Giardia lamblia* (0.53%; 5/941). Less common infections included *Echinostoma* spp. (0.11%; 1/941), *Ascaris lumbricoides* (0.11%; 1/941), *Enterobius vermicularis* (0.11%; 1/941), and the non-pathogenic amoeba *Entamoeba coli* (0.11%; 1/941) (Table 1).

**Table 1:** The prevalence of parasitic infections in participants from Ko-ae Subdistrict of Khueang Nai, Ubon Ratchathani Province.

Villages	Gender	Helminths					Protozoa		Mix infections					Total (%)
		OV	T	SS	HW	AL	GL	EC	OV, T	OV, HW	OV, SS	SS, EV	OV, SS, E	
Na mon	Male	–	2	5	–	–	–	–	–	–	–	–	–	7 (0.74%)
	Female	–	–	1	2	–	–	–	–	–	–	–	–	3 (0.32%)
Na Pho	Male	–	–	7	–	1	1	–	–	–	–	1	–	10 (1.06%)
	Female	–	–	2	–	–	–	–	–	1	–	–	–	3 (0.32%)
Tha Lat	Male	3	1	–	–	–	1	–	–	–	–	–	–	5 (0.53%)
	Female	2	–	1	1	–	–	–	–	–	–	–	–	4 (0.43%)
Dong Yang	Male	–	3	5	–	–	2	–	–	–	–	–	–	10 (1.06%)
	Female	1	1	4	–	–	–	–	–	–	–	–	–	6 (0.64%)
Yang Noi	Male	3	1	4	1	–	–	–	1	–	–	–	–	10 (1.06%)
	Female	3	–	–	–	–	–	–	–	–	–	–	–	3 (0.32%)
Ko	Male	3	–	–	–	–	–	–	–	–	–	–	–	3 (0.32%)
	Female	1	–	–	–	–	–	–	–	–	1	–	–	2 (0.21%)
Ae	Male	5	3	3	1	–	–	–	–	–	2	–	–	14 (1.49%)
	Female	1	2	1	–	–	–	1	–	–	1	–	–	6 (0.64%)
Pub	Male	10	–	–	–	–	–	–	–	–	–	–	–	10 (1.06%)
	Female	1	2	6	1	–	1	–	–	–	1	–	1	13 (1.38%)
Total (%)		33 (3.50%)	15 (1.59%)	39 (4.14%)	6 (0.63%)	1 (0.11%)	5 (0.53%)	1 (0.11%)	1 (0.11%)	1 (0.11%)	5 (0.53%)	1 (0.11%)	1 (0.11%)	109

**Abbreviations**: opisthorchiasis; OV, taeniasis; T, echinostomiasis; E, strongyloidiasis; SS, hookworm infection; HW, giardiasis; GL, ascariasis; AL, enterobiasis; EV, amoebiasis; EC.

**Fig. 1:**
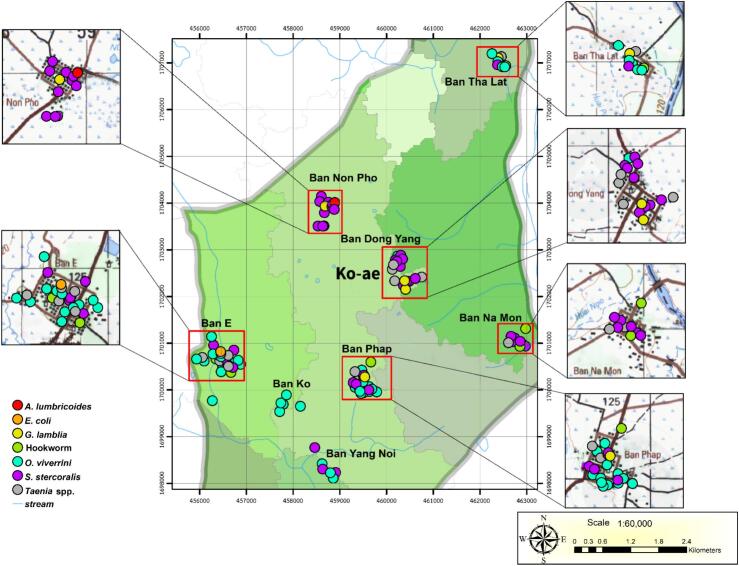
The distribution of parasitic infections in participants from eight villages of Ko-ae.

Seven cases of dual infections involving *O. viverrini* were recorded, including co-infections with *Taenia* spp. (*n* = 1), *S. stercoralis* (*n* = 5), and hookworm (n = 1). One case involved a co-infection of *S. stercoralis* and *E. vermicularis*. Additionally, one participant was found to have a triple infection involving *Echinostoma* spp., *O. viverrini,* and *S. stercoralis* (Table 1). All helminth infections, including opisthorchiasis, echinostomiasis, ascariasis, and hookworm, were classified as light infections (data not shown). The spatial distribution of PI among participants across the eight surveyed villages in Ko-ae is shown in Fig. 1, and representative microscopic images of helminth eggs and protozoan cysts detected among participants are shown in Fig. S1.

### 3.2 Socio-demographic characteristics and factors associated with PI

Participants diagnosed with and without PI were interviewed using a structured questionnaire to assess their socio-demographic profiles and risk factors for PI. Among the interviewees, male participants represented a higher proportion in both the infected (*n* = 58, 72.50%) and non-infected (*n* = 45, 56.25%) groups. The greater number of male respondents aligns with the higher prevalence of PI observed in males (*n* = 69, 63.30%) compared to females (*n* = 40, 36.70%). Logistic regression analysis indicated that male participants were significantly more likely to be infected with parasites than females, with an OR of 2.05 (95% CI: 1.06–3.97) (Table 2).

**Table 2:** Socio-demographic characteristics of participants diagnosed with and without parasitic infections.

Socio-demographic characteristics	Number of respondents infected with parasites (%)	Number of respondents non-infected with parasites (%)	OR	95%CI	*P*-value
Gender					
Male	58 (72.50)	45 (56.25)	2.05	1.06 to 3.97	0.033⁎
Female	22 (27.50)	35 (43.75)	1.00		
Age (year)					
20–30	10 (12.50)	40 (50.00)	1.00		
31–40	24 (30.00)	35 (43.75)	2.74	1.15 to 6.52	0.022⁎
41–50	30 (37.50)	5 (6.25)	24.00	7.42 to 77.57	< 0.001⁎
51–60	16 (20.00)	–	–	–	–
BMI					
Overweight and obesity	32 (40.00)	23 (28.75)	1.65	0.85 to 3.19	0.135
Underweight and normal weight	48 (60.00)	57 (71.25)	1.00		
Education					
Primary school	74 (92.50)	66 (82.50)	1.00		
Secondary/High school	6 (7.50)	14 (17.50)	0.38	0.13 to 1.05	0.063
Occupation					
Agriculture (rice field farmer)	66 (82.50)	56 (70.00)	1.00		
Employee	8 (10.00)	22 (27.50)	0.30	0.12 to 0.74	0.009⁎⁎
Merchant	6 (7.50)	2 (2.50)	0.84	0.11 to 6.21	0.872
Monthly income in Thai baht (USD)⁎⁎⁎					
<1000 (<32.05 USD)	42 (52.50)	13 (16.25)	1.00		
1001–5000 (32.08-160.26 USD)	34 (42.50)	62 (77.50)	0.16	0.08 to 0.36	<0.001⁎⁎
5001–10,000 (160.28-320.5 USD)	4 (5.00)	5 (6.25)	0.24	0.05 to 1.06	0.060
Comorbidity					
No	64 (80.00)	72 (90.00)	1.00		
Yes (Gout, Hypertension, Diabetes)	16 (20.00)	8 (10.00)	2.25	0.63 to 1.24	0.493

⁎ Statistically significant risk factor.

⁎⁎ Statistically significant protective factor.

⁎⁎⁎ Income was calculated based on the exchange rate on 3rd May 2021.

The age distribution of participants varied between groups. In the infected group, most respondents were between 41 and 50 years of age (*n* = 30, 37.50%), whereas in the non-infected group, the majority were aged 20–30 years (n = 40, 50.00%). The highest proportion of PI-positive participants fell within the 41–50 age range (n = 30, 37.50%) (Table 2), consistent with the overall prevalence data showing that the 41–50 age group had the highest rate of infection (*n* = 39, 35.78%) (Table S1). Furthermore, logistic regression analysis revealed a significant association between increasing age and risk of PI. Compared to respondents aged 20–30 years, those aged 31–40 and 41–50 years were 2.74 times (OR = 2.74; 95% CI: 1.15–6.52) and 24.00 times (OR = 24.00; 95% CI: 7.42–77.57) more likely to be infected, respectively (Table 2).

Health assessment data from the questionnaire indicated that the majority of participants in both groups had a normal body mass index (BMI), reported no comorbidities, and had completed only primary education. Statistical analysis showed that BMI, comorbidity status, and educational attainment were not significantly associated with the risk of PI (Table 2).

In addition, most respondents in both groups worked in agriculture, primarily as rice field farmers. However, participants employed in occupations with regular income had a statistically significant 70% lower risk of infection (OR = 0.30; 95% CI: 0.12–0.74) compared to those working in agriculture. Moreover, most respondents in Ko-ae reported poor economic status. In the infected group, the majority had a monthly income of less than 1000 baht (approximately 32.05 USD), whereas in the non-infected group, most had a monthly income between 1001 and 5000 baht (32.08–160.26 USD). Participants with a monthly income between 1001 and 5000 baht had a statistically significant 84% reduction in infection risk (OR = 0.16; 95% CI: 0.08–0.36) (Table 2).

### 3.3 Knowledge and perceptions related to PI

Respondents in both parasite-infected and non-infected groups were interviewed using ten statements designed to assess their knowledge and perceptions related to PI. Some statements were negatively keyed, meaning a false response was scored as correct, and the scoring was reversed accordingly.

The interview results indicated that most participants from both groups had limited knowledge and inaccurate perceptions, particularly regarding the efficacy of anthelmintic drugs. A majority believed that anthelmintic drugs could prevent parasitic infections. Such misconceptions may contribute to decreased attention to infection prevention and reluctance to undergo diagnostic testing for PI prior to chemotherapy.

The interview also revealed that most respondents from both groups were aware of the risk of *O. viverrini* infection through the consumption of raw or undercooked freshwater fish from endemic areas. They also recognized the association between opisthorchiasis and CCA. However, they demonstrated limited knowledge regarding the pathogenesis of CCA and other contributing risk factors, such as carcinogens present in fermented foods, which may promote the development of CCA in individuals with chronic opisthorchiasis.

Additionally, the interview indicated that all respondents in the infected group lacked knowledge about parasite transmission routes. Consequently, they were more likely to engage in unsanitary practices, including walking barefoot, handling food with unwashed hands, and consuming unwashed vegetables and fruits—behaviors that increase the risk of infection with foodborne parasites and soil-transmitted helminths. The interview further showed that none of the respondents in the infected group understood the potential health risks associated with using untreated human and animal feces as fertilizer, which can facilitate the spread of helminth eggs, larvae, and protozoan cysts into environmental reservoirs.

Finally, the analysis demonstrated that participants' knowledge and perceptions regarding PI and CCA were significantly associated with their PI status. Specifically, each one-point increase in the knowledge and perception score corresponded to a 31% reduction in infection risk (OR = 0.69; 95%CI: 0.57–0.84) (Table 3).

**Table 3:** Association between knowledge and perceptions regarding parasitic infections and CCA among parasite-infected and non-infected respondents.

Factors	Average score (SD)		OR	95%CI	*P*-value
	Parasite-infected group	Parasite non-infected group			
Knowledge and perceptions regarding parasitic infections	1.85 (1.32)	2.99 (2.21)	0.69	0.57–0.84	<0.001

## 4 Discussion

Ko-ae remains endemic for multiple PI. Despite ongoing urbanization that has converted some agricultural lands into residential and commercial areas, a large portion of the Ko-ae subdistrict still relies heavily on rice farming and agricultural labor, enabling continued exposure to parasite-contaminated environments.

Agricultural livelihood patterns could promote parasitic spread and cause rural communities to remain endemic to parasitic disease. The high prevalence of strongyliodiasis in Ko-ae that consistent with research conducted elsewhere in northeastern Thailand (Laoraksawong et al., 2018; Nuchprayoon et al., 2002; Sripa et al., 2025), reflecting persistent transmission driven by environmental exposure, agricultural practices, and socio-cultural behaviors (Aunpromma et al., 2016; Tesana et al., 2014). Furthermore, short rainy seasons and localized soil moisture retention in the region support the survival of *S. stercoralis* infective larvae, while habitual barefoot field work increases direct skin contact. In contrast, the lower prevalence of hookworm infection in Ko-ae aligns with the higher endemicity reported in southern Thailand, where rainfall and humidity levels are more favorable to hookworm survival (Sedionoto et al., 2019).

Mild intensity of opisthorchiasis is the second most common disease after strongyloidiasis in Ko-ae, despite longstanding national control measures (Kitphati et al., 2021). Food preferences and a deeply ingrained culture of raw food consumption prevent many inhabitants from changing their eating habits (Suwannahitatorn et al., 2019). Food sharing among households (Phimpraphai et al., 2018; Saenna et al., 2017) and poor preparation of Cyprinoid fish may sustain transmission risk.

The coexistence of soil-transmitted and food-borne helminths in Ko-ae highlights the simultaneous influence of environmental contamination and zoonotic reservoirs. Practices, such as the use of untreated fecal fertilizer, poor regulation of livestock (Ismail et al., 2010), abundance of stray animals (Aunpromma et al., 2016), and improper household waste management, all maintain the parasite life cycle in the environment (Pawestri et al., 2021).

Unsanitary behavioral norms can hinder compliance with prevention measures (Tesana et al., 2014). Therefore, environmental improvements, provision of protective equipment, regular stool screening, and anthelmintic treatment remain fundamental components of control strategies (Sripa et al., 2015). Additionally, raising awareness of CCA risk associated with chronic opisthorchiasis, supported by health education and liver ultrasound surveillance, is essential to reduce long-term morbidity in the community.

This study identified significant associations between PI and male sex (Table 2). These reflect social norms around masculinity and male gender occupation roles in agriculture in rural areas, increasing exposure to soil-transmitted helminths, subsequent social gatherings after farming, leading to increased exposure to food-borne parasites (Boonjaraspinyo et al., 2022; Chaiputcha et al., 2015; Phimpraphai et al., 2018; Wang et al., 2021), as reported in Thailand and other endemic settings such as Ethiopia and Laos (Gurmassa et al., 2024; Ribas et al., 2017). Furthermore, significant PI were observed in older age, specifically those aged 31–40 and 41–50 years (Table 2), suggesting long-term parasite exposure and declining immunity, which increases susceptibility to PI (Erwin and Maryanti, 2021; Jardim-Botelho et al., 2008).

While educational level, occupation type, and comorbidities showed limited statistical significance, the protective effect observed among employed individuals and those with moderate income highlights a social gradient in infection risk. However, low levels of educational attainment, particularly among parents, could promote high rates of PI in children due to parental less awareness, understanding of health risks, and engagement with healthcare services, impacting early detection, treatment, and parasite transmission cycles (Alharazi et al., 2020; Gizaw et al., 2019). Furthermore, poverty limits the ability of individuals or communities to prevent/control PI. Economic stability likely improves hygiene practices and access to sanitation, consistent with global evidence linking poverty reduction to decreased infection risk (Blackburn et al., 2023). However, most households in Ko-ae still fall below the average provincial income (∼620.80 USD/month), sustaining a cycle of poverty and socioeconomic vulnerability (The annual report on household income and income distribution of Ubon Ratchathani province, Statistical Office, Ubon Ratchathani Province, 2021).

Although BMI and comorbidity were not significantly associated with PI in this study, abnormal BMI and chronic diseases may impair immune defense, particularly in older adults (Cossa-Moiane et al., 2022; Jardim-Botelho et al., 2008; Kassalik and Mönkemüller, 2011; Rennie et al., 2021). In this study, BMI was examined in relation to overall PI, reflecting the real-world burden of polyparasitism in endemic settings. However, different parasite species exert distinct metabolic and nutritional consequences, and combining all species into a single outcome might dilute species-specific associations. Future analyses incorporating parasite-specific or infection-intensity-based stratification could improve mechanistic interpretation.

A notable finding was the discordance between knowledge and perceived protection from infection, particularly regarding overestimation of anthelmintic efficacy. Over-reliance on medication may reduce attention to hygiene, increase unsupervised self-medication, and impose financial burden, while potentially contributing to drug resistance (Boonjaraspinyo et al., 2022; Sripa et al., 2015). Application of the Knowledge–Attitude/Perception–Practice (KAP) framework should be used in this study to illustrate that knowledge alone is insufficient to drive behavioral change (Alharazi et al., 2020). Deep cultural values, taste preferences, and unsanitary habits continue to sustain risky behavior.

These findings highlight the importance of comprehensive, culturally grounded interventions. Integrated strategies, including health education, environmental sanitation improvements, regulated animal management, and supervised chemotherapy, implemented with strong community participation, are essential to achieving sustainable PI reduction in Ko-ae. However, future research on integrating environmental sampling (soil, fish, water) within a One Health framework would yield a more comprehensive understanding of transmission ecology and zoonotic linkages. Furthermore, evaluating the long-term impact of integrated programs will be critical for developing sustainable, scalable models for parasite control.

## 5 Conclusion

This survey represents the first report documenting the prevalence of PI and their associations with knowledge, attitudes/perceptions, practices, and other contributing factors among inhabitants of Ko-ae. A high prevalence of strongyloidiasis, followed by opisthorchiasis and other intestinal parasitic diseases, suggests that parasitic life cycles are actively maintained within environmental reservoirs in Ko-ae. In addition to unsanitary behaviors, male gender and age 31–40 and 41–50 years were identified as significant risk factors for PI. Although factors such as BMI, educational level, and comorbidities were not statistically associated with infection, they remain important considerations for comprehensive prevention strategies. Furthermore, residence in contaminated areas, poor socioeconomic status, agricultural occupations, limited accurate knowledge and perception of PI, and habitual engagement in unsanitary practices collectively contribute to the persistence of endemic parasitic transmission. These interrelated factors highlight the urgent need for coordinated efforts between local communities and public health agencies to deliver health services, implement targeted education programs, promote behavioral change, and ensure access to appropriate anthelmintic treatment for the residents of Ko-ae.

## CRediT authorship contribution statement

**Wirarat Jinatongthai:** Writing – original draft, Methodology, Investigation. **Phalakorn Suebsamran:** Validation, Software, Formal analysis, Data curation. **Rerkchai Srivoramas:** Visualization, Software, Formal analysis. **Tarinee Chaiwong:** Writing – review & editing, Methodology, Investigation. **Jittiyawadee Sripa:** Writing – review & editing, Validation, Supervision, Methodology, Data curation, Conceptualization.

## Funding

This research was supported by National Science, Research and Innovation Fund, and Ubon Ratchathani University, Research and Innovation Grant.

## Declaration of competing interest

The authors declare that they have no competing interests.

## Appendix A Supplementary data

Supplementary material Image 1
