# Supplementary material for: Parasite burden: Prevalence and risk factors in Ko-ae, Ubon Ratchathani, Thailand
Source: Parasite Epidemiol Control. 2026 Mar 2;33:e00491. doi: 10.1016/j.parepi.2026.e00491 (PMC12992520; doi:10.1016/j.parepi.2026.e00491)
Supplement: Supplementary file 1 — Supplementary material [file mmc1.docx]

**Supplementary data**

| **Age ranges** | **Males (%)** | **Females (%)** | **Total (%)** |
| --- | --- | --- | --- |
| 20-30 | 6 (5.50) | 7 (6.43) | 13 (11.93) |
| 31-40 | 19 (17.43) | 19 (17.43) | 38 (34.86) |
| 41-50 | 29 (26.61) | 10 (9.17) | 39 (35.78) |
| 51-60 | 15 (13.76) | 4 (3.67) | 19 (17.43) |
| **Total (%)** | 69 (63.30) | 40 (36.70) | 109 |

**Table S1** The parasitic infections in participants in each age range.

**Table S2** The questionnaire survey on knowledge and perceptions associated with parasitic infections and CCA.

| **Knowledge and perceptions regarding parasitic infections and CCA** | **Parasite-infected group** | | | **Parasite non-infected group** | | |
| --- | --- | --- | --- | --- | --- | --- |
|  | **True (%)** | **False (%)** | **Do not know (%)** | **True (%)** | **False (%)** | **Do not know (%)** |
| Q1. Anthelmintic drugs can prevent parasitic infections. | 68 (85.00%) | 6 (7.50%) | 6 (7.50%) | 48 (60.00%) | 18 (22.50%) | 14 (17.50%) |
| Q2. Anthelmintic drugs are generally safe to use. | 10 (12.50%) | 22 (27.50%) | 48 (60.00%) | 15 (18.75%) | 10 (12.50%) | 55 (68.75%) |
| Q3. It is unnecessary to know the status of parasitic infection before taking anthelmintic drugs. | 12 (15.00%) | 14 (17.50%) | 54 (67.50%) | 5 (6.25%) | 30 (37.50%) | 45 (56.25%) |
| Q4. Consuming raw freshwater fish from the endemic area could be at risk of liver fluke infection. | 60 (75.00%) | 12 (15.00%) | 8 (10.00%) | 66 (82.50%) | 5 (6.25%) | 9 (11.25%) |
| Q5. Bile duct cancer can be caused by a liver fluke. | 46 (57.50%) | 14 (17.50%) | 20 (25.00%) | 55 (68.75%) | 5 (6.25%) | 20 (25.00%) |
| Q6. Consuming fermented foods could increase the risk of cancer. | 0 | 0 | 80 (100%) | 10 (12.50%) | 12 (15.00%) | 58 (72.50%) |
| Q7. Walking barefoot could put one at risk of infection with hookworm. | 0 | 0 | 80 (100%) | 15 (18.75%) | 13 (16.25%) | 52 (65.00%) |
| Q8. The use of human and animal feces as fertilizer can increase the spread of intestinal helminth and protozoa infective stages into environmental reservoirs. | 0 | 0 | 80 (100%) | 15 (18.75%) | 8 (10.00%) | 57 (71.25%) |
| Q9. Washing fresh vegetables and fruits thoroughly before eating can reduce the risk of intestinal helminth and protozoan infections. | 0 | 0 | 80 (100%) | 10 (12.50%) | 9 (11.25%) | 61 (76.25%) |
| Q10. Washing hands before eating can reduce the risk of infection with intestinal helminths and protozoa. | 0 | 0 | 80 (100%) | 10 (12.50%) | 13 (16.25%) | 57 (71.25%) |


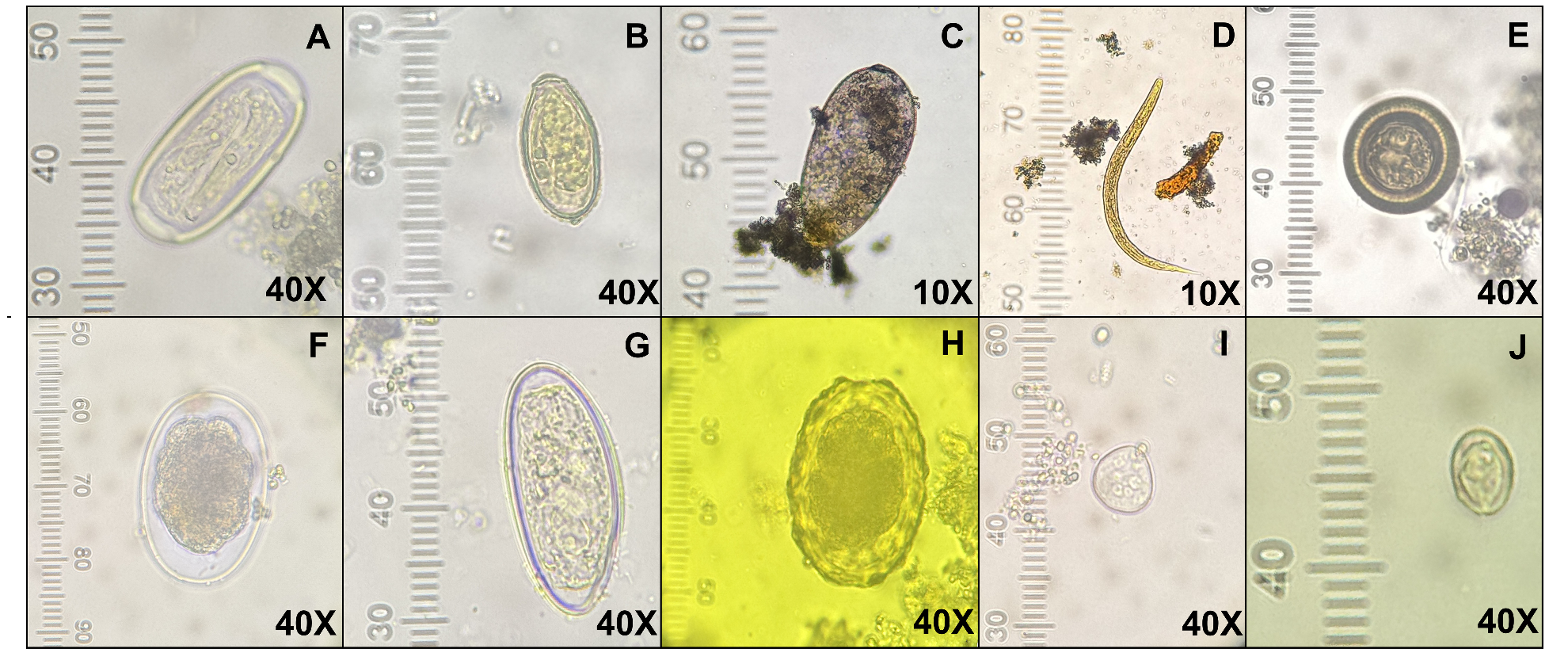


**Figure S1** Representative microscopic images of helminth eggs and protozoan cysts detected among participants. Light micrographs showing characteristic morphological features of helminth eggs and protozoan cysts observed in stool samples from participants. The specimens illustrate diagnostic stages of different parasites identified in the Ko-ae community, including *T. trichiura* egg (A), *O. viverrini* egg (B), *Echinostoma* spp. egg (C), *S. stercoralis* rhabdifiform larvae (D), *Taenia* spp. egg (E), hookworm egg (F), *E. vermicularis* egg (G), *A. lumbricoides* fertilized egg (H), *E. coli* cyst (I), and *G. lamblia* cyst (J). Images were captured using an Euromex microscope equipped with HD-Pro HDMI camera (Arnhem, The Netherlands).
